# Draft Genome Sequences of Two Cellulolytic *Paenibacillus* sp. Strains, MAEPY1 and MAEPY2, from Malaysian Landfill Leachate

**DOI:** 10.1128/genomeA.00065-14

**Published:** 2014-02-13

**Authors:** Patric Chua, Hye-Seung Yoo, Han Ming Gan, Sui-Mae Lee

**Affiliations:** School of Science, Monash University, Selangor, Malaysia

## Abstract

We report the draft genome sequences of two *Paenibacillus* species with cellulose-degrading abilities isolated from landfill leachate. An array of genes putatively involved in cellulose degradation have been identified in both genome sequences, which can benefit various biotechnological industries.

## GENOME ANNOUNCEMENT

In landfills, the majority of wastes are organic in nature and are represented by plant components, such as cellulose and hemicellulose. These materials are fairly resistant to environmental degradation and yet have been proven to be the only source of food for the landfill bacterial community. The process by which cellulosic materials are degraded into simple units of carbon and nitrogen requires a complete suite of cellulolytic enzymes, including exoglucanases, endoglucanases, and β-glucosidases, to work in unison (1, 2). Much interest is focused on cellulose-degrading bacteria and their enzymes.

*Paenibacillus* sp. strains MAEPY1 and MAEPY2 are Gram-positive, spore-forming bacteria isolated from landfill leachate samples acquired from the Jeram Sanitary Landfill, Selangor, Malaysia. Both were isolated due to their ability to degrade both amorphous and crystalline forms of cellulose. They were cultured in Sizova’s minimal salt medium using carboxymethyl cellulose (CMC) as the sole carbon source (3). Genomic DNA was isolated using the GF-1 nucleic acid extraction kit (Vivantis, Malaysia). Based on 16S rRNA gene analyses, MAEPY1 and MAEPY2 formed a monophyletic group with bacterial strains in the genus *Paenibacillus.*

Genome sequencing was performed using the Illumina MiSeq (150-bp paired-end reads). The raw reads were trimmed and assembled *de novo* using CLC Genomics Workbench 6 (CLC bio, Denmark), producing 183 contigs with an accumulated length of 7,478,507 bp (53-fold coverage; N_50_, 189,001 bp) for MAEPY1 and 129 contigs with an accumulated length of 7,470,797 bp (64-fold coverage; N_50_, 219,342 bp) for MAEPY2. Both strains have a DNA G+C content of 45%. Using the Rapid Annotations using Subsystems Technology (RAST) gene annotation, tRNAscan 1.2, and RNAmmer 1.2 (4–6), 6,800 open reading frames (ORFs), 93 tRNAs, and 4 rRNAs, respectively, were predicted from the draft genome of strain MAEPY1 and 6,799 ORFs, 103 tRNAs, and 5 rRNAs, respectively, were predicted from the draft genome of MAEPY2. Only 34% of the ORFs in each strain were assigned putative functions according to the subsystem categorization.

Cellulolytic gene clusters were predicted from the draft genomes of both strains using the SEED comparative genomics resource and InterPro (7, 8). Thirteen genes encoding enzymes putatively involved in cellulose degradation, such as endoglucanase (EC 3.2.1.4), exoglucanase (EC 3.2.1.91), and β-glucosidase (EC 3.2.1.21), were identified in both strains. Further analysis of the sequences subsequently grouped the enzymes into 6 different glycoside hydrolase families, families 1, 3, 5, 8, 26, and 48.

### Nucleotide sequence accession numbers.

The draft genome sequences of *Paenibacillus* sp. MAEPY1 and MAEPY2 have been deposited at DDBJ/EMBL/GenBank under accession no. AWUJ00000000 and AWUK00000000, respectively.
